# Availability of PPEs and training status of health professionals on COVID-19 in Silte Zone, Southern Ethiopia

**DOI:** 10.11604/pamj.2021.39.38.27648

**Published:** 2021-05-13

**Authors:** Mohammed Muze, Bahredin Abdella, Abdilmejid Mustefa, Abas Ali, Abdulfeta Abdo, Abas Lukman, Abdulfeta Shafi, Shukure Uomer, Yesufe Badege, Abdulmejid Mutteba, Bayesa Tolasa, Sister Hossae, Sultan Shukur, Ebrahim Ahmed, Abdu Kemal, Tadela Erena

**Affiliations:** 1 Department of Nursing, College of Medicine and Health Sciences, Werabe University, Worabe, Southern Ethiopia,; 2 Department of Political Science, College of Social Science and Humanities Werabe University, Worabe, Southern Ethiopia,; 3 Department of Statistics, College of Natural and Computation Sciences, Werabe University, Worabe, Southern Ethiopia,; 4 Department of Biology, College of Natural and Computation Sciences, Werabe University, Worabe, Southern Ethiopia,; 5 Department of Animal Science, College of Agriculture and Natural Resource, Werabe University, Worabe, Southern Ethiopia,; 6 Department of Mathematics, College of Natural and Computation Sciences, Werabe University, Worabe, Southern Ethiopia,; 7 Department of English, College of Social Science and Humanities, Werabe University, Worabe, Southern Ethiopia,; 8 Department of Physics, College of Natural and Computation Sciences, Werabe University, Worabe, Southern Ethiopia,; 9 Department of Agro Economics, College of Agriculture and Natural Resource, Werabe University, Worabe, Southern Ethiopia

**Keywords:** Personal protection equipment, COVID-19, training, Ethiopia

## Abstract

**Introduction:**

recent infectious disease outbreaks like COVID-19 highlights the importance of personal protective equipments and competent professionals on public health preparedness and response in health care systems. Hence, understanding availability of personal protective equipments and training status of health professionals is very important to fill the gap of COVID-19 preparedness and response. Therefore, this study was conducted to assess availability and adequacy of personal protective equipments and health professional's training status on COVID-19 in Silte Zone, southern Ethiopia.

**Methods:**

cross sectional study was conducted from August to October 2020 in Silte Zone. First four weredas from 13 were selected randomly. Systematic sampling technique was used to select 351 health professionals from 13 health facilities of selected weredas.

**Results:**

overall, only 36.1% of the health professionals have received adequate training on COVID-19. About 30% of the health professionals had taken training on emergency plan of COVID-19, about 33% had been taught on COVID-19 treatment procedures. Majority 80.9% of the respondents indicated that personal protective equipments are inadequately available. Face masks, hand sanitizers and eye goggles were most scarce PPEs.

**Conclusion:**

health professionals have been at the frontlines in responding to the COVID-19 pandemic. Yet, challenges remain, such as limited availability of personal protection equipments and inadequate training of healthcare professions was identified by this study. Strengthening of training on COVID-19 and making PPEs adequately available were recommended.

## Introduction

Coronavirus disease (COVID-19) is an infectious disease caused by a newly discovered coronavirus. The disease was first identified in December 2019 in Wuhan, the capital of Hubei province of China, and now quickly spreading around the world, resulting in the ongoing coronavirus pandemic [1]. To date, globally, more than 78.8 million cases have been reported across 218 countries and territories, resulting in more than 1,733,488 deaths. More than 55,452,004 people have recovered [2]. Most people infected with the COVID-19 virus will experience mild to moderate respiratory illness and recover without requiring special treatment. Older people and those with underlying medical problems like diabetes, cardiovascular disease, chronic respiratory disease, and cancer are more likely to develop severe illness. The virus spreads primarily through droplets of saliva or discharge from the nose when an infected person sneezes or coughs. While the pandemic in Africa is several weeks behind Europe and Asia, the number of cases in Africa is escalating fast [3-5].

Healthcare workers are more susceptible to COVID-19 infection than the general population due to frequent contact with infected individuals. Additionally, some procedures such as non-invasive ventilation, high-flow nasal cannula and bag-mask ventilation may generate higher aerosol volumes [6,7]. The World Health Organization reported that a one in ten health worker is infected with coronavirus in some countries. Its recommendations include the training of healthcare workers and providing adequate personal protective equipment to decrease the spread of COVID-19 [8]. Personal protective equipment (PPE) includes gowns, gloves, face masks, and a face shield or goggles. Risks for infection may also be highest at the beginning of the outbreak when healthcare workers may not yet be familiar with PPE use. There are major PPE shortages in high-income countries and it is likely that limited supplies will be allocated to less resourced countries [9-11]. In resource limited area most limited PPEs need to be identified to prioritize the allocation.

Beside of personal protective equipment, training and capacity building has been a critical pillar in this recent outbreak. Most countries have nowadays national response, inter-country and regional response mechanisms that require to be complemented by international assistance in case of crises. Therefore, the humanitarian community needs an adequate pool of dedicated, qualified and experienced public health professionals who are properly trained to appropriately and sufficiently support these countries during the critical phases of a major emergency, disaster or humanitarian crisis. These are professionals who are not only technically equipped with the best practices and tools in public health in emergencies, but also with management and operational skills required in the delivery of coordinated, integrated and equitable public health action in challenging, rapidly- changing, chaotic and sometimes insecure environments [12]. Assessing the training needs of local public health workers is an important step toward providing appropriate training programs in emergency preparedness. It has been well established that most public health agency employees need training in core public health competency. Many public health agency workers enter the public health workforce with training in only their specific technical area [13,14]. To the best of our knowledge, there has never been study carried out in Ethiopia evaluating the availability of PPEs and training status of health professionals on COVID-19. Therefore main aim of this study was to assess the above issues.

## Methods

**Study design, period and setting:** institution based cross sectional study was conducted from August to October 2020 in Silte Zone. It is one of fourteen zones of the southern region of Ethiopia and found 172km away from Addis Ababa (the capital city of Ethiopia). Based on last census conducted by the central statistical agency of Ethiopia, in 2018 the Zone has estimated population of 1,017,557. There are 4 hospitals and 33 health centers. There were also 1800 health professionals.

**Study subjects:** the source population of this study was health professionals working in health facilities of Silte zone. Selected health professionals were the study population.

**Sample size determination and sampling technique:** the sample size (n) required for the study was calculated using a single population proportion formula. Considering the lack of similar study conducted at similar context as per our search effort, we took p-value of 50%, 5% marginal error, and 95% confidence interval were used to obtain the maximum sample size. Initial sample size was 384. With the addition of 10% contingency, correction of our sample size, initial sample size was decreased from 384 to 351. First, four weredas from 13 were selected randomly. Three hundred fifty one health professionals working in all health facilities of selected weredas were selected by systematic sampling technique.

**Data collection tools and quality control:** self-administered questionnaire was used to collect the data adopted from literature. Training status was assessed by 7 questions adopted from previous literature. All of the questions have yes or no answer. Mean is used as a cut off value to categorize training status into adequate or inadequate [15]. To control the quality of data, trained supervisors supervise the data collection and check the completeness of the questionnaire on the daily base. Data was entered into a computer using Epi data version 3.1, and edited, cleaned and analyzed using SPSS version 20. Descriptive statistics were used to describe the data.

**Ethical consideration:** permission letter was obtained from Werabe University before data collection was initiated. It was submitted to Silte Zone administrative bodies to obtain their co-operation. The purpose of the study was explained to the study subjects. At the time of data collection, an informed consent was taken from the participants to confirm whether they are willing to participate. Those not willing to participate were given the right to do so. Confidentiality of responses was also ensured throughout the research process.

## Results

**Socio-demographic characteristics of study participants:** a total of 351 questionnaires were distributed and 324 were returned, which gives an overall 92% response rate. From total of the study respondents, 186 (57.4%) were male. Of all the respondents, 130 (40.1%) were single and 181 (55.9%) were married. Regarding educational level, about 150 (46.3%) of the respondents were diploma followed by 147(45.4%) BSc degree. The mean age of the study participants was 26.51±3.4 years (Table 1).

**Table 1:** socio-demographic characteristics of study participants in Silte Zone, Southern Ethiopia, 2020

Variables	Category	Frequency	Percent (%)
Sex	Male	186	57.4
	Female	138	42.6
	Total	324	100.0
Marital status	Single	130	40.1
	Married	181	55.9
	Others	13	4.0
	Total	324	100.0
Educational level	Diploma	150	46.3
	BSc	147	45.4
	GP	8	2.5
	Others	19	5.9
	Total	324	100.0
Age	Minimum	Maximum	Mean/Std
	19	45	26.51±3.4

**Training status of the health professionals:** overall, only 36.1% of the health professionals have received adequate training on COVID-19. About 30% of the health professionals had taken training on emergency plan of COVID-19, 32.4% had been taught on COVID-19 treatment procedures, and 49.8% had been taught Methods of identifying suspected case of COVID-19. More than half (60.2%), of the health professionals received training on awareness of COVID-19. About half (50.3%) of the health professionals had been taught personal protective measures. Less than half 38.6%, 42% and 35.8% information system management of COVID-19, disinfection and sterilization and principles of quarantine and isolation respectively (Table 2).

**Table 2:** training status of health professionals in Silte Zone, southern Ethiopia, 2020

S.no	Training status	Response	Frequency	Percent
1	Content of emergency plan	No	228	70.4
		Yes	96	29.6
		Total	324	100.0
2	COVID -19 treatment procedures	No	219	67.6
		Yes	105	32.4
		Total	324	100.0
3	Methods of identifying suspected case of COVID	No	166	51.2
		Yes	158	48.8
		Total	324	100.0
4	Awareness of COVID -19	No	129	39.8
		Yes	195	60.2
		Total	324	100.0
5	Personal protective measures	No	161	49.7
		Yes	163	50.3
		Total	324	100.0
6	Information system management of COVID 19	No	199	61.4
		Yes	125	38.6
		Total	324	100.0
7	Disinfection and sterilization	No	188	58.0
		Yes	136	42.0
		Total	324	100.0
8	Principles of quarantine and isolation	No	208	64.2
		Yes	116	35.8
		Total	324	100.0
9	Over all training status	Poor	207	63.9
		Good	117	36.1

**Protective equipment availability:** regarding protective equipment availability, majority of the respondents indicated that availability of masks, sanitizers and surgical gloves were 89.2%, 76.9% and 67.9% respectively. But most of the respondents did not use masks in work place. They also responded on availability of other protective equipments below the average in health facility which included disposable full-face shields 33.6%, eye goggles 41.4%, and disposable gloves 42.3%. However, majority 262 (80.9%) of the respondents indicated that PPEs were inadequately available; hand sanitizers, facemasks, and eye goggles were most scarce PPEs.

## Discussion

Training of health professionals was an opportunity to improve performance and enhance the quality of service. The COVID-19 crisis has stressed the importance of training front line health workers [16]. Our findings can be used to identify training needs and scarce PPEs. According to our study, only one third (36.1%) of the health professionals have received adequate training on COVID-19. The finding is comparable with a finding of a study done in Yemen where 41% of health professionals had ever been trained on public health emergency [17]. Possible explanation to have a low training activity in the study area is due to lack of adequate budget to COVID-19 interventions. At the early stage of COVID-19 response and readiness there may be uncertainty of allocating budget to specific interventions; concerned body may face a difficulty of prioritizing interventions leads to have limited training activities. Current study also observed the detail of the training, about half and above of health professionals had been taught methods of identifying suspected case of COVID-19, awareness of COVID-19 and personal protective measures whereas less than half had taken training on emergency plan of COVID-19, COVID-19 treatment procedures, information management system of COVID-19, disinfection and principles of quarantine. The finding indicates the trainings given in the study area lack uniformity and is inadequate. Training is key elements of emergency preparedness [18]. Having inadequate training may leads to high rate of COVID-19 infections among health professionals. Additionally, it may leads to difficulty in controlling COVID-19 transmission in the study area. Thus, strengthening the training of health professionals on COVID-19 is necessary.

The present study also assessed the availability and adequacy of PPEs. Respondents reported the availability of protective equipment like disposable full-face shields, gown, eye goggle and disposable glove in the health facilities. However, majority of the respondents indicated that PPEs are inadequately available; hand sanitizers, facemasks, and eye goggles are most scarce PPEs. Another study also reported the shortage of PPEs of COVID-19 [19]. Based on the available evidence, the COVID-19 virus is transmitted between people through close contact and droplets. The people most at risk of infection are those who are in close contact with a COVID-19 patient or who care for COVID-19 patients [20]. So that making PPEs are adequately available to health professionals is very important to control transmission of COVID-19 among health professionals.

**Limitations:** the lack of similar studies hindered further comparison of the results with other regions or different groups in the country; however, this study forms the basis upon which comparisons with other studies may be made.

## Conclusion

The finding of this study indicates one third of health professionals had received adequate training on COVID-19. Majority of the respondents reported that PPEs are inadequately available; hand sanitizer, face mask, and eye goggle are most scarce PPEs. Strengthening the training of health professionals on COVID-19 and Making PPEs adequately available are recommended.

**Funding:** the study was funded by the Werabe University and the funder only involved on funding, not take a part in design of the study, data collection, analysis, and interpretation.

### What is known about this topic

- COVID-19 is pandemic diseases;
- Personal protective equipment are very important to control transmission of COVID-19;
- Training of health workers is essential to control of COVID-19 transmission.

### What this study adds

- Status of COVID-19 training among health professionals is poor;
- Personal protective equipment are inadequately available;
- Most scarce PPEs are identified.
